# A Fearless Critic and a Master of Humorous Expression

**Published:** 1919-09-13

**Authors:** 


### CHARLES ARTHUR MERCIER,

### M.D., F.R.C.P.Lond., F.R.C.S.Eng.

### A Fearless Critic and a Master of Humorous Expression.

The death of Dr. Mercier leaves a gap in the medical profession which may not easily be filled. The statement is trite and conventional if considered merely from the aspect of his scientific capabilities, and he was not one who had any great toleration for the trite and conventional. But from another point of view it is incontrovertible—namely, when he is regarded as a fearless critic, an attacker of shams and pseudo-philosophies, and a master of humorous expression.

His early days were spent as a sailor and then as an employee in a woollen warehouse. However much he may have been influenced by his life at sea, there can be no doubt that the woollen warehouse left little impress in his personality or on that reflection of it, his literary style. His periods were anything but woolly. A more appropriate occupation would have been something to do with steel: he had all the incisiveness of the knife-edge and the probing power of the rapier.

His academic career was so brilliant that he might have entertained doubts as to his success in later life! If so his fears were not well founded. Of course, if one contemplates life from the point of view of the Smilesian, the question may be debated. If, however, success means to have filled a worthy and honourable place in the community, success he indubitably had.

Much of his time was spent in actual charge of the insane, first, in county asylums—the Bucks County Asylum and the City of London Asylum at Stone—and later as resident physician at Plower House, Catford. This was not, however, the aspect of his professional work which appealed chiefly to his interest, although he wrote a useful volume on asylum construction and administration. His bent was much more towards theory and exposition. In this direction he was influenced by the teachings of Herbert Spencer and of that great thinker, Hughlings Jackson. Unrestrained by feelings of fallibility he became a prolific writer. "Sanity and Insanity," a volume he wrote for the Contemporary Science Series in 1890, is still probably the most readable account of the subject and may fittingly be placed beside Clouston's "Hygiene of Mind." "Psychology, Normal and Morbid," is a more detailed and more technical statement of his views. But he looked far afield and dealt at length with subjects cognate to the study of mental disorder. His writings on criminology—a term he disliked—are well-known: and on two occasions he won the Swiney Prize, first for his "Criminal Responsibility," and, a few months ago, for "Crime and Criminals." He laid about him vigorously when discussing the views of other criminologists, Lombroso, in particular, being subjected to the weight of his blows. Here as elsewhere, however, his impatience of opinions contrary to his own led him to do less than justice to the work of others, and he was apt to be dogmatic where knowledge, still inchoate, does not yet allow of dogmatism.

The be-all and end-all in the study of insanity was for Mercier—taken *au grand serieux*—conduct, how the patient acted. He ended by almost convincing himself that this was what he believed. From the legal point of view this was well enough, but what the scientific mind is looking for is the cause—cerebral disorder, for example—of the variations in conduct. It seems a logical conclusion: and Mercier, who was himself a logician—at least he assailed the logic of others from Aristotle onwards—probably realised it. But too many others held to that opinion for it to be immune from his onslaughts.

It is in his more polemical writings that his prominent characteristics appear. There is something of Aristophanes, not a little of Pope, and a good deal of Swift, in his way of looking at things. Sometimes there is the pardonable exaggeration—to take examples from another art—of Rowlandson or of Gilray. Satire and mordant humour, occasionally passing into broad farce, are to be found in his *obiter dicta* on spiritualism, on psycho-analysis, on alcohol, and on the opposition to forcible feeding. Sometimes it appeared as if he were first setting up men of straw and then attacking them: but, big and large, his criticisms were well directed and often more effective than the ponderous weight of more academic attacks. Withal there was the saving grace of humour.

No account of his career would be complete without a reference to the work which he did towards providing for the treatment of incipient insanity. He was also an active member on various committees of the Medico-Psychological Association, and was at one time its President. The Medico-Legal Society was also beholden to him; and other learned societies have had the advantage of his assistance.

His latter days were a struggle against ill-health and physical discomfort. His nervous energy triumphed over these and enabled him to continue with his literary work practically up to the time of his death. It is said that he was even projecting a new book. There would have been words of cold comfort in it, one may surmise, for psycho-analysis, for spiritualism, or for the German school of alienists!

